# The association between serum lipid levels and histological type of breast cancer

**DOI:** 10.1186/s40001-022-00784-y

**Published:** 2022-08-19

**Authors:** Xinru Wang, Yajie Wang, Miaomiao Wang, Xin Chen, Wenjing Cui, Xiao Chen

**Affiliations:** 1 Department of Radiology, the Affiliated Hospital of Nanjing University of Chinese Medicine, 155 Hanzhong road, Nanjing, 210029 China; 2 Department of Radiology, Shantou Central Hospital, 114 Waima road, Shantou, 515041 China; 3 Department of Radiology, Shanghai Sixth People’s Hospital, Shanghai, 200233 China; 4 Institute of Radiation Medicine, Fudan University Shanghai Medical College, Shanghai, 200032 China

**Keywords:** Breast cancer, Histologic type, Blood lipids

## Abstract

**Background:**

Studies have investigated the association between serum lipids level or apolipoprotein levels and breast cancer (BC) risk. However, the relationship between serum lipids level and apolipoprotein levels and histological type of breast cancer remains unclear. This study was aimed to explore the association between serum lipids level and the histological type of BC, particularly to estrogen receptor (ER) and progesterone receptor (PR) positive BC.

**Materials and methods:**

220 cases of pathology-confirmed BC were retrospectively collected in this study. Patients’ demographic information, clinical data, and pathological features were obtained from medical records. Serum levels including high-density lipoprotein-cholesterol (HDL-c), low-density lipoprotein-cholesterol (LDL-c), total cholesterol (TC), triglyceride (TG), apolipoprotein A (ApoA), ApoB, ApoE and lipoprotein a(LP(a)) were collected before treatment. Logistic regression analyses were used to show the association between serum lipids and subtypes of BC. Receiver operating characteristic (ROC) curves were generated to analyze the predictive performance.

**Results:**

There were 70 ER-negative and 73 PR-negative BC. Patients with ER-negative BC had higher HDL-c, higher LDL-c, and higher LP(a) than those in ER-positive one (*p* < 0.05). Patients with PR-negative BC were more likely to have high LDL-c and high LP(a) levels than patients with PR-positive one (*p* < 0.05). Multivariate logistic regression analysis showed that serum HDL-c (odds ratio (OR): 0.27, 95% confidence interval (CI) 0.10–0.76), LDL-c (OR: 0.19, 95%CI 0.04–0.93) and LP(a) (OR: 0.23,95%CI 0.07–0.80) levels were negatively associated with ER-positive BC, and serum HDL-c and LDL-c levels were significantly negatively associated with PR-positive BC (OR: 0.32, 95%CI 0.12–0.82; OR: 0.14, 95%CI 0.03–0.77). In addition, ER and PR positive BC was negatively associated with serum HDL-c and LDL-c levels (OR = 0.39, 95% CI 0.17–0.91; OR = 0.22, 95% CI 0.06–0.85) after adjusting with confounders. Serum HDL-c level (OR = 0.13, 95% CI 0.02–0.87) was still independently associated with ER and PR positive BC in postmenopausal women. The area under the curves (AUCs) of HDL-c to identify ER-positive BC, PR-positive BC, and ER and PR positive BC were 0.65 (95%CI 0.58–0.73, *P* < 0.01), 0.62 (95%CI 0.54–0.69, *P* < 0.01) and 0.64 (95%CI 0.56–0.72, *P* < 0.01), respectively.

**Conclusions:**

Serum HDL-c and LDL-c levels were related to ER or PR positive BC. Lipid levels may also have acceptable performance in identifying BC histological type.

## Introduction

Breast cancer (BC) is the second common cause of death in women. In 2021, 281,550 new cases of BC occurred, which corresponds to 30% of all new cancer cases in women worldwide [1]. It’s important to determine the expression status of estrogen receptor (ER), progesterone receptor (PR), and HER2 in BC, because they are associated with prognosis, endocrine therapies, and adjuvant treatment decisions [2, 3]. ER and/or PR negative BC are more aggressive and have worse prognosis than ER and/or PR positive one. Comprehensive strategies are urgently needed to change the profiles of the BC burden.

Lipid metabolism is reprogrammed in tumors [4]. Reprogramming cholesterol metabolism in endocrine-related cancers is related to cell proliferation, migration and invasion, which could be potentially available for future hormonal therapy [5, 6]. Serum lipids and lipoproteins may have direct effects on tumor proliferation and migration [7–9]. The associations between lipids levels and cancer risk were also reported by several epidemiological studies [10, 11]. Cholesterol level is associated with lung cancer risk and outcomes [12, 13]. A recent study showed that triglycerides (TG)/high-density lipoprotein (HDL) ratio may have potential in identifying bladder cancer [4]. Low serum HDL levels are also associated with malignant behavior of pancreatic neuroendocrine neoplasms [14]. In addition, studies also showed that oxidized low-density lipoprotein-cholesterol (LDL-c), or high total serum cholesterol (TC) and HDL-cholesterol (HDL-c) was associated with more aggressive cancer [15, 16].

Obesity and hypercholesterolemia are both potentially associated with breast cancer risk [17]. The association between serum lipids levels and BC risk had been studied. Some clinical studies showed an inverse association between blood levels of TC, HDL-c and BC risk [18]. A positive association between blood levels of LDL and BC risk were also reported [19]. Moreover, some studies showed that the use of statin might be associated with decrease mortality of BC patients [20, 21]. Recently, a study showed that HDL-c levels were associated with malignant intraductal papillary mucinous neoplasms [22] which indicated that HDL may be related to more malignant histological type of neoplasms. The biological behavior of BC was also related to its histological type. However, the relationship between serum levels of lipid and apolipoprotein and histological type of BC has not been thoroughly investigated. In the present study, the association between serum lipid level and the histological type of BC, particularly to ER and PR positive BC, was observed.

## Materials and methods

### Patients

220 patients with biopsy proven unilateral primary BC during 2016–2019 in Affiliated Hospital of Nanjing University of Chinese Medicine were included in our study after excluding by these criteria:(1) patients below 20 years; (2) receiving hormone replacement therapy; (3) without complete blood lipids data; and (4) administration of hypolipidemic drugs within 1 month. Patients’ demographic information, clinical data, and pathological features were obtained from medical records. The laboratory data including serum HDL-c, LDL-c, TC, TG, apolipoprotein A (ApoA), ApoB, ApoE and lipoprotein(a) (LP(a)) were collected within 1 month of diagnosis. This retrospective study was approved by the Institutional Ethic Review Board of the Jiangsu Province Hospital of Chinese Medicine. Informed consent was waived because of the retrospective design. Declaration of Helsinki were adhered during the study.

### Serum lipid determination

Blood samples were collected from each patients after at least 8 h of fasting in coagulant-coated tubes. HDL-c, LDL-c, TC, TG, ApoA, ApoB, ApoE and LP(a) were determined in a fully automatic biochemical analyzer. We also calculated the ratio of HDL/TC, LDL/TC, ApoA/ApoB, HDL/ApoA and LDL/ApoB.

### Histological examinations

Immunohistochemical markers of estrogen receptor (ER) (*n* = 220), progesterone receptor (PR) (*n* = 217) were also collected from pathological records. Positive ER and PR expression were considered if greater than 1% of the tumor cells exhibited nuclear staining. Three cases did not have information of PR expression. The BC were classified into molecular subtypes: ER positive, PR positive, and ER + PR positive. WHO grade for BC was also evaluated: Grade I, well-differentiated; Grade II, moderately differentiated; Grade III, poorly differentiated.

### Statistical analysis

Receiver-operating curve (ROC) were generated and the cutoff values for serum HDL-c, LDL, TC, TG, ApoA, ApoB, ApoE, LP(a), HDL/TC, LDL/TC, ApoA/ApoB, HDL/ApoA and LDL/ApoB were calculated using the Youden index (sensitivity + specificity − 1). The two-tailed t test (data with normal distribution) or Mann–Whitney U test (data with abnormal distribution) was used to compare the continuous variables, while the chi-square test was used to compare the categorical variables. Spearman correlation analysis was used to show the correlation among variables. Univariate and multivariate logistic regression analyses were used to show the association between the blood lipids levels and the histological subtypes. ROC curves were generated to analyze predictive performance of lipid levels. All statistical analyses were performed using SPSS 20.0 (IBM Corp., Armonk, NY, USA). Sample sizes were estimated using PASS (version 2021). Test for two proportions was used (power = 0.80, *α* = 0.05). The estimated difference in the prevalence of low HDL-c between ER positive and ER negative BC was 17%. The estimated sample size was 224. Our sample size was close to the estimated one. *P* values less than 0.05 were considered statistically significant.

## Results

### The characteristics of subjects

A total of 220 women were included in this study, and the characteristics of subjects are listed in Table 1. There were 70 ER-negative and 73 PR-negative BC. The long diameters of ER or PR negative BC were larger than ER or PR positive BC, respectively (*P* < 0.05). Low grade of lesion (WHO I&II) were more common in ER or PR positive BC (*P* < 0.001). History of miscarriage was more common in women with ER-positive BC (*P* = 0.03). However, no significant differences were found in other characteristics, such as age, body mass index and BC family history.

**Table 1:** Characteristics of patients according to the expression status of estrogen receptor (ER) and progesterone receptor (PR)

Variables	ER expression status			PR expression status		
	ER-BC (*N* = 70)	ER + BC (*N* = 150)	*P*	PR-BC (*N* = 73)	PR + BC (*N* = 144)	*P*
Age (years)	70	150	0.82	73	144	0.39
Less than50	31 (44.3%)	64 (42.7%)		29 (39.7%)	66 (45.8%)	
50 and more	39 (55.7%)	86 (57.3%)		44 (60.3%)	78 (54.2%)	
Height (cm)	160.2 ± 3.1	159.7 ± 4.9	0.53	159.1 ± 4.7	160.0 ± 4.7	0.18
Weight (kg)	62.0 ± 7.5	61.8 ± 8.5	0.90	60.8 ± 8.7	61.9 ± 8.5	0.36
BMI	70	149	0.15	73	143	0.35
Normal	40 (57.1%)	100 (67.1%)		42 (57.5%)	95 (66.4%)	
Overweight	28 (40.0%)	41 (27.5%)		28 (38.4%)	41 (28.7%)	
Obesity	2 (2.9%)	8 (5.4%)		3 (4.1%)	7 (4.9%)	
Menopausal state	70	149	0.35	72	144	0.44
Premenopause	39 (55.7%)	73 (49.0%)		40 (55.6%)	72 (50.0%)	
Postmenopause	31 (44.3%)	76 (51.0%)		32 (44.4%)	72 (50.0%)	
Hypertension	70	149	0.10	72	144	0.60
Yes	15 (21.4%)	48 (32.2%)		19 (26.4%)	43 (29.9%)	
No	55 (78.6%)	101 (67.8%)		53 (73.6%)	101 (70.1%)	
Diabetes	70	149	0.11	72	144	0.87
Yes	3 (4.3%)	16 (10.7%)		6 (8.3%)	13 (9.0%)	
No	67 (95.7%)	133 (89.3%)		66 (91.7%)	131 (91.0%)	
Coronary heart disease	70	149	0.18^a^	72	144	0.67^a^
Yes	0	5 (3.4%)		1 (1.4%)	4 (2.8%)	
No	70 (100%)	144 (96.6%)		71 (98.6%)	140 (97.2%)	
BC family history	70	149	0.24^a^	72	144	0.26^a^
Yes	2 (2.9%)	1 (0.7%)		2 (2.8%)	1 (0.7%)	
No	68 (97.1%)	148 (99.3%)		70 (97.2%)	143 (99.3%)	
Parity	61	129	0.49^a^	63	125	0.61^a^
Nulliparous	2 (3.3%)	7 (5.4%)		4 (6.3%)	5 (4.0%)	
Once or twice	57 (93.4%)	112 (86.8%)		56 (88.9%)	111 (88.8%)	
More than twice	2 (3.3%)	10 (7.8%)		3 (4.8%)	9 (7.2%)	
Miscarriage history	53	117	0.03	54	114	0.18
Non	25 (47.2%)	34 (29.1%)		24 (44.4%)	35 (30.7%)	
Once or twice	25 (47.2%)	63 (53.8%)		25 (46.3%)	61 (53.5%)	
More than twice	3 (5.7%)	20 (17.1%)		5 (9.3%)	18 (15.8%)	
Location	70	150	0.61	73	144	0.70
Left	39 (55.7%)	78 (52.0%)		40 (54.8%)	75 (52.1%)	
Right	31 (44.3%)	72 (48.0%)		33 (45.2%)	69 (47.9%)	
Long diameter	67	148	< 0.01	70	142	0.02
≤ 5 cm	51 (76.1%)	133 (89.9%)		54 (77.1%)	127 (89.4%)	
> 5 cm	16 (23.9%)	15 (10.1%)		16 (22.9%)	15 (10.6%)	
WHO grade	64	129	< 0.001	66	127	< 0.001
I&II	28 (43.8%)	98 (76.0%)		29 (43.9%)	97 (76.4%)	
III	36 (56.3%)	31 (24.0%)		37 (56.1%)	30 (23.6%)	
Pathological type	70	150	0.46^a^	73	144	0.43^a^
Tis	5 (7.1%)	10 (6.7%)		5 (6.8%)	8 (5.6%)	
Invasion, NST	64 (91.4%)	132 (88.0%)		67 (91.8%)	129 (89.6%)	
Invasion, ST	1 (1.4%)	8 (5.3%)		1 (1.4%)	7 (4.9%)	

^a^Fisher’s exact test

ER + BC: ER-positive breast cancer; ER-BC: ER-negative breast cancer; PR + BC: PR-positive breast cancer; PR-BC: PR-negative breast cancer;

### Serum lipid levels

Significance differences were observed in the levels of serum HDL-c, LDL-c and LP between ER-positive and ER-negative BC (*P* < 0.05). The similar results were found in PR-positive and PR-negative BC (Table2). In addition, LDL/TC and LDL/ApoB were significantly associated with PR-positive BC (*P* < 0.05), but no significant differences were found in HDL/TC, ApoA/ApoB and HDL/ApoA.

**Table 2:** Serum lipids level of patients according to the expression status of estrogen receptor (ER) and progesterone receptor (PR)

Variables	ER expression status			PR expression status		
	ER-BC (*N* = 70)	ER + BC (*N* = 150)	*P*	PR-BC (*N* = 73)	PR + BC (*N* = 144)	*P*
HDL-c (mmol/L)	70	150	0.02	73	144	0.28
≤ 1.36	16 (22.9%)	58 (38.7%)		21 (28.8%)	52 (36.1%)	
> 1.36	54 (77.1%)	92 (61.3%)		52 (71.2%)	92 (63.9%)	
LDL-c (mmol/L)	70	150	0.03	73	144	< 0.01
≤ 2.08	5 (7.1%)	28 (18.7%)		4 (5.5%)	29 (20.1%)	
> 2.08	65 (92.9%)	122 (81.3%)		69 (94.5%)	115 (79.9%)	
TC(mmol/L)	70	150	0.24	73	144	0.36
≤ 5.12	48 (68.6%)	114 (76.0%)		51 (69.9%)	109 (75.7%)	
> 5.12	22 (31.4%)	36 (24.0%)		22 (30.1%)	35 (24.3%)	
TG(mmol/L)	70	150	0.09	73	144	0.30
≤ 1.52	57 (81.4%)	106 (70.7%)		57 (78.1%)	103 (71.5%)	
> 1.52	13 (18.6%)	44 (29.3%)		16 (21.9%)	41 (28.5%)	
ApoA(g/L)	70	150	0.28	73	144	0.61
≤ 1.34	39 (55.7%)	95 (63.3%)		43 (58.9%)	90 (62.5%)	
> 1.34	31(44.3%)	55 (36.7%)		30 (41.1%)	54 (37.5%)	
ApoB(g/L)	70	150	0.15	73	144	0.08
≤ 0.76	17 (24.3%)	51 (34%)		17 (23.3%)	50 (34.7%)	
> 0.76	53 (75.7%)	99 (66%)		56 (76.7%)	94 (65.3%)	
ApoE(mg/dL)	70	150	0.17	73	144	0.71
≤ 4.7	47 (67.1%)	114 (76.0%)		52 (71.2%)	106 (73.6%)	
> 4.7	23 (32.9%)	36 (24.0%)		21 (28.8%)	38 (26.4%)	
LP(a) (mg/L)	70	150	0.02	73	144	0.01
≤ 33	11 (15.7%)	45 (30%)		11 (15.1%)	44 (30.6%)	
> 33	59 (84.3%)	105 (70%)		62 (84.9%)	100 (69.4%)	
HDL/TC	70	150	0.25	73	144	0.56
≤ 0.29	18 (25.7%)	50 (33.3%)		21 (28.8%)	47 (32.6%)	
> 0.29	52 (74.3%)	100 (66.7%)		52 (71.2%)	97 (67.4%)	
LDL/TC	70	150	0.21	73	144	0.04
≤ 0.64	42 (60.0%)	103 (68.7%)		41 (56.2%)	101 (70.1%)	
> 0.64	28 (40.0%)	47 (31.3%)		32 (43.8%)	43 (29.9%)	
ApoA/ApoB	70	150	0.62	73	144	0.87
≤ 1.22	19 (27.1%)	36 (24%)		19 (26.0%)	36 (25%)	
> 1.22	51 (72.9%)	114 (76%)		54 (74.0%)	108 (75%)	
HDL/ApoA (mmol/g)	70	150	0.25	73	144	0.24
≤ 1.09	21 (30%)	57 (38%)		22 (30.1%)	55 (38.2%)	
> 1.09	49 (70%)	93 (62%)		51 (69.9%)	89 (61.8%)	
LDL/ApoB	70	150	0.16	73	144	0.04
(mmol/g)	11 (15.7%)	14 (9.3%)		13 (17.8%)	12 (8.3%)	
≤ 2.66	59 (84.3%)	136 (90.7%)		60 (82.2%)	132 (91.7%)	

ER + BC:ER-positive breast cancer; ER-BC: ER-negative breast cancer; PR + BC: PR-positive breast cancer; PR-BC: PR-negative breast cancer;

*HDL-c* high-density lipoprotein-cholesterol, *LDL-c* low-density lipoprotein-cholesterol, *TC* total cholesterol, *TG* triglyceride, *ApoA* apolipoprotein A, *ApoB* apolipoprotein B, *ApoE* apolipoprotein E, *LP* lipoprotein

### Correlation analysis

Based on receiver operating curve (ROC) analysis, the recommended cutoff values were 1.36 mmol/L for HDL-c, 2.08 mmol/L for LDL-c, 5.12 mmol/L for TC, 1.52 mmol/L for TG, 1.34 mmol/L for ApoA, 0.76 mmol/L for ApoB, 4.7 mmol/L for ApoE, 33 mmol/L for LP(a), 0.29 for HDL/TC,0.64 for LDL/TC, 1.22 for ApoA/ApoB, 1.09 mmol/g for HDL/ApoA and 2.66 mmol/g for LDL/ApoB. Then, we divided lipid levels into categorical variables based on the above cutoff value.

**Fig. 1:**
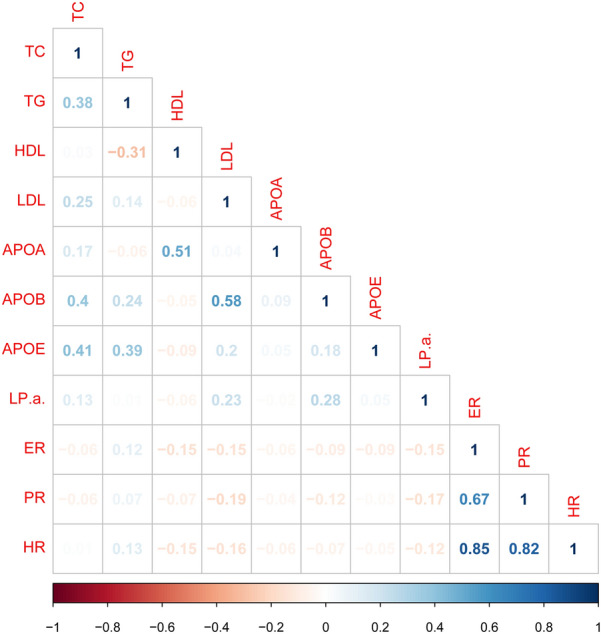
The spearman correlation between the different levels of serum lipids and histological type of breast cancer. *R* < − 0.135 or *r* > 0.135, *P* < 0.05

The Spearman correlation analysis are shown in Fig. 1. The categorical data was used in the analysis. The HDL-c was positively associated with the ApoA (*r* = 0.51, *P* < 0.001). In addition, statistical significance was found the levels of TC, LDL-c and ApoB (*P* < 0.001). LDL-c and Lp(a) were correlated to ER or PR expression (*P* < 0.05). HDL-c was correlated with ER or ER + PR expression (*P* < 0.05).

### Univariate and multivariate logistic regression

Univariate and multivariate logistic regression analyses was used to identify the associated factors for ER-positive BC and PR-positive BC (Table 3). For ER-positive BC, the odds ratio (OR) value was 0.45 (95%CI 0.21–0.95) for serum HDL-c level, 0.43 (95% CI 0.14–1.29) for serum LDL-c level, and 0.57 (95% CI 0.25–1.31) for serum LP(a) level. After adjusting with age, body mass index (BMI) and miscarriage history, these factors were still independently associated with ER positive BC (OR = 0.27, 95% CI 0.10–0.76; OR = 0.19, 95% CI 0.04–0.93; OR = 0.23, 95% CI 0.07–0.80). For PR positive BC, the levels of serum HDL-c and LDL shown an independent association after additionally adjusting with confounding factors (OR = 0.32, 95% CI 0.12–0.82; OR = 0.14, 95% CI 0.03–0.77). Moreover, WHO grade was significant associated with positive ER (OR = 0.11, 95%CI: 0.04–0.30), PR (OR = 0.15, 95%CI 0.06–0.38) and ER + PR expression (OR = 0.23, 95%CI 0.11–0.47).

**Table 3:** Univariate and multivariate logistic regression to the estrogen receptor (ER)-positive breast cancer (BC) and progesterone receptor (PR)-positive BC

Variables	ER-positive BC			PR-positive BC		
	Univariate	Multivariate		Univariate	Multivariate	
	OR(95%CI)	Model 1 OR(95%CI)	Model 2 OR(95%CI)	OR(95%CI)	Model 1 OR(95%CI)	Model 2 OR(95%CI)
HDL-c (≤ 1.36 vs > 1.36)	0.47 (0.25–0.90)	0.45 (0.21–0.95)	0.27 (0.10–0.76)	0.71 (0.39–1.32)	0.70 (0.34–1.45)	0.32 (0.12–0.82)
LDL-c (≤ 2.08 vs > 2.08)	0.34 (0.12–0.91)	0.43 (0.14–1.29)	0.19 (0.04–0.93)	0.23 (0.08–0.68)	0.31 (0.10–1.01)	0.14 (0.03–0.77)
LP(a) (≤ 33.0 vs > 33.0)	0.43 (0.21–0.44)	0.57 (0.25–1.31)	0.23 (0.07–0.80)	0.40 (0.19–0.84)	0.47 (0.20–1.09)	0.37 (0.12–1.16)
Long diameter (≤ 5 cm vs > 5.0 cm)	0.46 (0.23–0.92)	0.35 (0.14–0.86)	0.21 (0.06–0.72)	0.47 (0.23–0.95)	0.40 (0.16–0.96)	0.35 (0.11–1.08)
WHO grade (I&II vs III)	0.22 (0.13–0.40)	0.21 (0.11–0.42)	0.11 (0.04–0.30)	0.25 (0.14–0.44)	0.21 (0.11–0.42)	0.15 (0.06–0.38)

Model 1 included HDL, LDL, LP, Long diameter and WHO grade;

Model 2 was additionally adjusted with age, body mass index and miscarriage history

Categorical variables were used in logistic analyses

*HDL-c* high-density lipoprotein-cholesterol, *LDL-c* low-density lipoprotein-cholesterol, *LP* lipoprotein

Subsequently, we showed the association between ER and PR positive BC and the levels of serum HDL-c and LDL-c (Table 4). After additionally adjusting with age and BMI, HDL-c and LDL-c were associated with the presence of ER and PR positive BC (OR = 0.39, 95% CI 0.17–0.91; OR = 0.22, 95% CI 0.06–0.85). Furthermore, we found that serum HDL-c (OR = 0.13, 95% CI 0.02–0.87) was still independently associated with ER and PR positive BC in postmenopausal women (Table 5).

**Table 4:** Univariate and multivariate logistic regression to the estrogen receptor (ER) and progesterone receptor (PR) positive breast cancer

Variables	Univariate		Multivariate			
			Model 1		Model 2	
	OR (95%CI)	*P*	OR (95%CI)	*P*	OR (95%CI)	*P*
WHO grade (I&II vs III)	0.24 (0.13–0.43)	< 0.001	0.23 (0.11–0.47)	< 0.001	0.23 (0.11–0.47)	< 0.001
Long diameter(≤ 5 cm vs > 5.0 cm)	0.56 (0.23–1.17)	0.12	0.50 (0.20–1.25)	0.14	0.52 (0.21–1.31)	0.17
HDL-c(≤ 1.36 vs > 1.36)	0.46 (0.23–0.94)	0.03	0.49 (0.21–1.16)	0.047	0.39 (0.17–0.91)	0.03
LDL-c(≤ 2.08 vs > 2.08)	0.25 (0.07–0.87)	0.03	0.28 (0.08–1.04)	0.05	0.22 (0.06–0.85)	0.03
TC (≤ 5.12 vs > 5.12)	1.06 (0.53–2.13)	0.87				
TG (≤ 1.52 vs > 1.52)	2.15 (0.98–4.74)	0.06				
ApoA (≤ 1.34 vs > 1.34)	0.76 (0.41–1.41)	0.39				
ApoB (≤ 0.76 vs > 0.76)	0.70 (0.35–1.40)	0.32				
LP(a) (≤ 33 vs > 33)	0.49 (0.22–1.09)	0.08				
ApoE (≤ 4.7 vs > 4.7)	0.78 (0.40–1.53)	0.47				
HDL/TC (≤ 0.29 vs > 0.29)	0.53 (0.26–1.08)	0.08				
LDL/TC (≤ 0.64 vs > 0.64)	0.66 (0.35–1.23)	0.19				
ApoA/ApoB(≤ 1.22 vs > 1.22)	0.67 (0.32–1.41)	0.29				
HDL/ApoA (≤ 1.09 vs > 1.09)	0.54 (0.27–1.06)	0.07				
LDL/ApoB (≤ 2.66 vs > 2.66)	2.18 (0.91–5.18)	0.08				

Model 1 included HDL, LDL, long diameter and WHO grade; Model 2 was additionally adjusted with age and body mass index

*HDL-c* high-density lipoprotein-cholesterol, *LDL-c* low-density lipoprotein-cholesterol, *TC* total cholesterol, *TG* triglyceride, *ApoA* apolipoprotein A, *ApoB* apolipoprotein B, *ApoE* apolipoprotein E, *LP* lipoprotein

**Table 5:** Multivariate logistic regression to the estrogen receptor (ER) and progesterone receptor (PR)-positive BC for postmenopausal women

Variables	Multivariable			
	Model 1		Model 2	
	OR (95%CI)	*P*	OR (95%CI)	*P*
HDL-c(≤ 1.36 vs > 1.36)	0.14 (0.03–0.76)	0.02	0.13 (0.02–0.87)	0.04
LDL-c(≤ 2.08 vs > 2.08))	0.29 (0.03–2.83)	0.28	0.29 (0.03–3.37)	0.33
WHO grade (I&II vs III)	0.13 (0.04–0.44)	0.001	0.09 (0.02–0.36)	0.001
Long diameter(≤ 5.0 vs > 5.0)	0.60 (0.15–2.46)	0.48	0.83 (0.15–4.52)	0.83

Model 1 included HDL, LDL, long diameter and WHO grade;

Model 2 was additionally adjusted with age and body mass index

*HDL-c* high-density lipoprotein-cholesterol, *LDL-c* low-density lipoprotein-cholesterol;

Categorical variables were used in logistic analyses

### ROC analysis

**Fig. 2:**
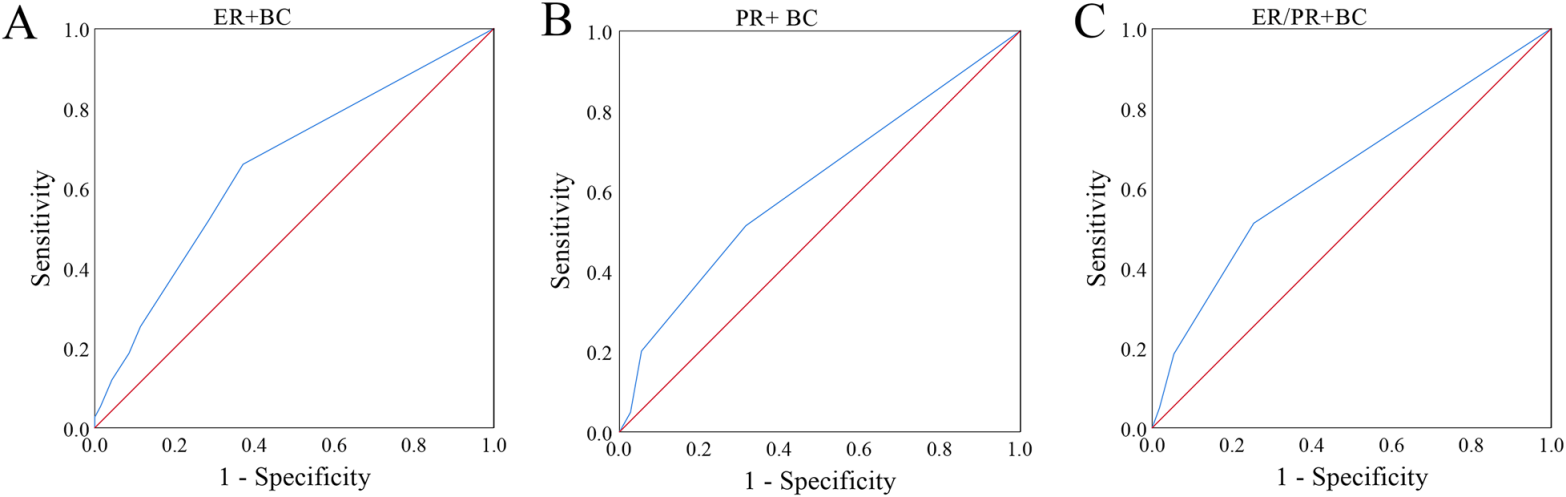
The receiver operating characteristic (ROC) curves of lipids in predicting breast cancer (BC) histological type. A: high-density lipoprotein-cholesterol (HDL-c), low-density lipoprotein-cholesterol (LDL-c) and lipoprotein (a) (LP(a)) in predicting estrogen receptor (ER)-positive breast cancer (ER + BC). B: HDL-c and LDL-c in predicting progesterone receptor (PR)-positive breast cancer (PR + BC). C: HDL-c and LDL-c in predicting ER and/or PR positive breast cancer (ER/PR + BC)

The performance of serum HDL-c, LDL-C and Lp(a) in identifying ER or PR expression is shown in Fig. 2. The area under the ROC curve (AUC) of serum HDL-c + LDL-c + LP(a) in identifying ER positive BC was 0.65 (95%CI 0.58–0.73, *P* < 0.01). The positive likelihood ratio (PLR) and negative likelihood ratio (NLR) were 1.78 and 0.54, respectively. Moreover, the AUCs of serum HDL-c plus LDL-c in identifying PR positive BC and ER/PR positive BC were 0.62 (95%CI 0.54–0.69, *P* < 0.01) (PLR and NLR were 1.63 and 0.71, respectively) and 0.64 (95%CI 0.56–0.72, *P* < 0.01) (PLR and NLR were 2.01 and 0.65, respectively), respectively.

## Discussion

ER and PR expression status in BC are associated with therapy and clinical prognosis. In the present study, we also observed that both ER-positive and PR-positive BC are negatively associated with worse prognostic characteristics, such as larger lesion and higher WHO grade. Clinical evidence for the association between the lipids level and the ER/PR expression status of BC has not been thoroughly investigated. The present study showed that low HDL-c and LDL-c were independently associated with ER-positive and PR-positive BC. Lipid levels also have potential to predict ER/PR expression in BC.

HDL-c and breast cancer risk have been investigated in different countries or regions. Most of the studies demonstrated an inverse correlation between HDL-c and breast cancer risk, disease free survival (DFS) or overall survival (OS) [23]. A meta-analysis also supported an association between HDL-c and BC risk [18]. Studies also showed the mechanism of HDL on cancer development or progression [24]. HDL-associated proteins may enhance anti-tumorigenesis effects by exerting their biological activities, such as antioxidant, anti-inflammatory, anti-angiogenesis, and immunomodulatory [25, 26]. In addition, high level of scavenger receptor type B-I (SR-BI) expression was involved in lipid internalization and lipoprotein consumption, which results in reducing HDL-c levels during cancer [24, 27]. We inferred that low HDL-c may positively associated with aggressive BC type. However, we found that the HDL level was negatively associated with ER or PR positive BC in the present study. The mechanisms are not clear. The cholesterol metabolism should be considered during the growth of BC. We speculated that ER or PR negative BC shows high proliferation which need more cholesterol. Then HDL is increased to transfer more cholesterol to BC cells besides liver cells. Moreover, some studies supported our results. An in vitro study showed that HDL stimulated proliferation in both ER-positive and ER-negative BC cell lines in a dose-dependent manner, but ER-negative cells showed a higher response [19]. A prospective study investigated the relationship between HDL-c and ER/PR positive BC, and showed a significant inverse association (HR = 0.45, 95% CI 0.21–0.97) [28]. However, a Mendelian randomization study showed that genetically raised HDL-c is associated with higher risk of ER-positive BC(OR = 1.13,95%CI 1.01–1.26) [29]. HDL-c levels was not only generically determined, but also environmentally associated. BC cells may modify the progress of lipid metabolism.

Some studies showed that LDL-c is not associated with breast cancer risk, while serum LDL level might be a predictor of BC progression [30–32]. However, it was demonstrated that significant upregulation of LDL receptor increased LDL uptake in cancer cells because of the demand of rapid proliferation. Two Mendelian randomization studies found that genetically elevated plasma LDL level appeared to be associated with increased BC risk [27, 33]. In addition, LDL treatment promoted breast cells viability, and enhanced tumor progression and migration [26]. LDL promoted ER-negative cells proliferation such as MDA-MB-231 and MDA-MB-436 faster than ER-positive cells such as MCF-7 [34]. This study found that serum LDL level was reverse associated with ER or PR positive BC. It seemed that increased LDL might be associated with more malignant breast cancer phenotype. Interestingly, a study also indicated that higher levels of LDL at diagnosis was associated with high proliferative tumors, with higher grade, and with advanced stages [30]. However, the underlying mechanism has not been clarified. Few studies have shown the association between LDL-c levels and BC histological types. This association may be also related to cholesterol metabolism. LDL promotes transport of cholesterol from liver to the tumor cells [35]. ER or PR negative BC may need more cholesterol. Therefore, the LDL-c levels increased in ER or PR negative BC. LDL receptor was found to be upregulated in triple negative BC [34, 36], which also suggested that the more malignant the BC is, the more their need for LDL.

ApoA was a major protein component of HDL-c. Few studies have identified the role of ApoA in breast cancer. An analyses performed on 7,557 subjects in France showed that ApoA was inversely associated with the breast cancer risk (HR = 0.36, 95% CI: 0.18–0.73) [9]. One study showed that serum ApoA level was an independent prognostic factor in invasive ductal breast cancer [37]. A recent study showed that ApoA or ApoE stimulated tumor growth in MCF-7 cells (ER-positive cells) and inhibit tumor ability in MDA-MB-231 cells (ER-negative cells) [38]. The present study found no significant association between ApoA or ApoE levels with BC histological type. In addition, ApoB is a major protein component of LDL-c. Few studies showed the association between ApoB and BC. One study found that breast cancer risk was inversely associated with ApoB (HR = 0.92, 95%CI 0.86–0.99) [39]. To the best of our knowledge, no study showed the association between serum ApoB levels and BC histological type. The present study showed a weak association between ApoB or LDL/ApoB and PR expression. Those data supported that BC histological type was also associated to cholesterol in HDL-c or LDL-c. More studies are needed to evidence such correlation.

The microenvironment of fluctuating lipid-metabolic conditions may affect BC phenotype [40]. Borgquist. et al. [41] found that cholesterol-lowering medication may have a role in preventing breast cancer recurrence in ER/PR positive early stage BC, which showed an interaction of serum lipids with estrogen-sensitive breast tissues. In fact, many cholesterol-derived metabolites such as 27-hydroxycholesterol (27HC) can promote cellular adaptation by altering cholesterol targets during BC development. As an endogenous selective ER modulator (SERM), 27HC exhibited sufficient estrogenic activity to support the proliferation of ER-positive BC cells [17, 19, 42, 43], which might contribute to the identification of drug-targets and the design of novel therapies in BC patients.

This study has several limitations. First, the sample size was relatively small, even though it was close to the estimated one. Second, it would be better to collect multiple lipid measures besides baseline. This study may fail to show the dynamic correlation during the tumor development. Third, this study only observed the phenomenon between lipid levels and BC histological type. The underlying mechanisms in cell/mouse model levels were not investigated. Fourth, the correlation between lipid serum levels with clinical parameters like overall and.

progression-free survival was not studied. In addition, this is a retrospective study, selection bias cannot be avoided. Finally, some confounding factors were not considered, such as lifestyle and diet habits.

In conclusion, this study shows that low HDL-c and LDL-c are associated with ER-negative and PR-negative BC. Lipid levels might be associated with BC phenotype. Intervention on lipid may be a potential strategy for the treatment of ER or PR-negative BC. Lipid levels may also have acceptable performance in identifying BC histological type. Further studies are needed to confirm this association and explore the possible mechanism.

## Data Availability

All data generated or analyzed during this study are available from the corresponding author on reasonable request.

## Abbreviations

AUC: Area under the curve
BC: Breast cancer
CI: Confidence interval
HDL-c: High-density lipoprotein-cholesterol
LDL-c: Low-density lipoprotein-cholesterol
TC: Total cholesterol
TG: Triglyceride
ApoA: Apolipoprotein A
ApoB: Apolipoprotein B
ApoE: Apolipoprotein E
LP(a): Lipoprotein(a)
OR: Odds ratio
ROC: Receiver operating characteristic
BMI: Body mass index
